# Nylon fiber waste as a prominent adsorbent for Congo red dye removal

**DOI:** 10.1038/s41598-023-51105-0

**Published:** 2024-01-11

**Authors:** Kareem H. Hamad, Ahmed M. Yasser, Radwa Nabil, Raneem Tarek, Eslam Hesham, Ahmed El-telbany, Ahmed Saeed, Salah E. Selim, Ahmed E. Abdelhamid

**Affiliations:** 1 Egyptian Academy for Engineering and Advanced Technology (EA&EAT) Affiliated to Ministry of Military Production, Cairo, Egypt; 2 Polymers and Pigments Department, National Research Centre, 33 El-Buhouth St., Dokki, 12622 Giza Egypt

**Keywords:** Environmental sciences, Chemistry, Engineering

## Abstract

In this research nylon fibers wastes (NF) were fabricated into porous sheet using a phase inversion technique to be utilized as an adsorbent materials for Congo red dye (CR). The fabricated sheet denoted as NS was characterized using FTIR and XRD. The surface studies of the adsorbent materials using SEM and BET analysis reveals a highly pores structure with an average pore volume 0.61 cc/g and BET surface area of 767 m^2^/g. The adsorption studies of fabricated NS were employed into CR at different parameters as pH, effect of time and dye concentration. The adsorption isotherm and kinetic studies were more fit to Langmuir and pseudo second order models. The maximum adsorption capacity q_max_ reached 188 mg/g with removal percentage of 95 for CR concentration of 400 mg/L at pH 6 and 0.025 g NS dose for 10 ml CR solution. The regeneration study reveals a prominent adsorption behavior of NS with removal % of 88.6 for CR (300 mg/L) after four adsorption desorption cycles. Effect of incorporation of NaonFil Clay to NS was studied using Response Surface Methodology (RSM) modeling and reveals that 98.4% removal of CR could be achieved by using 19.35% wt. of fiber with 8.2 g/L dose and zero clay, thus at a predetermined parameters studies of NanoFil clay embedded into NS, there are no significant effect for %R for CR.

## Introduction

Because wastewater contains so many industrial and organic pollutants, there are now significant environmental problems across the globe. These organic pollutants are produced from a variety of sources, including residential sewage, agricultural runoff, industrial effluents and medical waste^1^. The wastewater containing the organic contaminants contains a wide range of synthetic chemicals, including phenols, industrial compounds, insecticides, detergents, oils, and fertilizers^2,3^. These substances are exceedingly poisonous and harm both aquatic life and human health. Synthetic dyes are the most prevalent organic contaminants in wastewater and are released into the environment because of faulty dyeing and treatment procedures. Dye effluents come from various industries, including textile, leather, paper, and plastics^4^. As previously, reported, the industries companies immediately release a significant amount (up to 15%) of dye-polluted effluent into water bodies; this amount is estimated to be between 70 and 200 thousand tons annually. The presence of dyes in aquatic environments causes a reduction in the amount of dissolved oxygen and sunlight penetration, strangling aquatic plants and creatures and interfering with their ability to photosynthesize. The textile industry uses cationic, anionic, and non-ionic synthetic dyes^5^.

Dyes are extremely hazardous and have carcinogenic properties due to their poisonous nature, high solubility in water, limited degradability, and complicated structural compositions. even at low doses, affects humans and animals^6,7^. In this regard, numerous approaches, such as biological, ion exchange, photo catalytic oxidation, coagulation-flocculation, membrane filtration, and adsorption treatments, have been reported for the removal of dyes from wastewater before their release into the environment^8–12^. The usage of most of these technologies have been restricted as a result of their flaws, which include high operational costs, residual waste creation, constrained processing capacity, and complex procedures^13,14^.

Comparatively, it has been revealed that one of the best techniques for treating water is the adsorption procedure. This is due to the fact that this approach stands out for its straightforward design, cost effectiveness, simplicity in use and handling, high efficiency, and accessibility to a variety of adsorbent materials^15,16^. Numerous adsorbent substances, including polymeric composite adsorbents, metallic and metal organic frameworks, natural and synthetic polymers, agricultural biomass and their composites, activated carbon, clay with variant types, e.g. zeolite and cloisite and their composites, silica and ion exchange polymer, have been employed^17–24^.

The fuzzes and fibers trash that the textile industry produces is another type of materials that can have bad effects on the environment. Researchers have therefore tried to utilize fiber waste in different applications, such as soil reinforcement material and the adsorption process, but only after chemical treatment^25^. One of the most abundant textile fiber waste are nylon 66 and nylon 6. Because the chains lack aromatic components, they have a tendency to fold, resulting in fibers with a low modulus and a relatively high extension at break. However, the amide groups allow the NH and CO groups of adjacent chains to establish hydrogen bonds, which provides the fiber great mechanical and thermal stability. The amide groups also make the fiber more water-attractive (4% moisture recovery), which makes it more wettable^26^. Few researches were employed nylon waste as a prominent material used for water treatment. Recently, the copper removal adsorption capacity for nylon/chitosan composite was found to be 28 mg/g, but after mixing algal biomass, the adsorption capacity considerably increased to 35.86 mg/g. Freundlich modelling was successfully obeyed to remove the chosen micro pollutants^27^. The fabricated nylon-6-polyaniline nanocomposites was successfully adsorb up to 370 mg/g methyl orange as an anionic dye^28^. In another research, polyamide 66 nanofiber modified citric acid was prepared via electrospinning technique from nylon fiber waste, the adsorption capacity was investigated for methylene blue dye and it was found to be 27.93 mg/g^29^.

In this study, we focus on fabrication of porous nylon sheet from nylon fiber waste via phase inversion techniques and study the performance of the resulted sheet as a candidate adsorbent material for Congo red dye. In addition, the effect of incorporation of Nanofil clay on adsorption performance on nylon sheet was studied by using response surface methodology modeling technique (RSM).

## Experimental

### Materials

**Figure 1:**
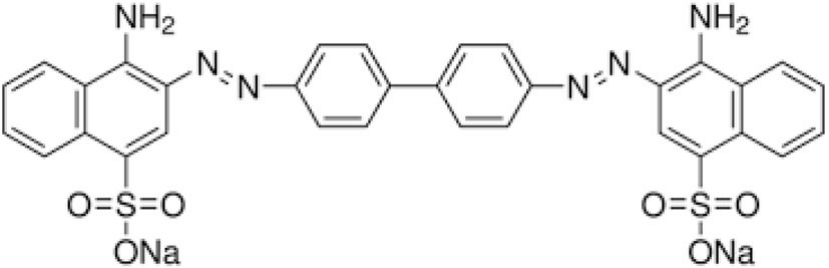
Congo red dye chemical structure.

Nylon fiber waste consisting mainly of nylon 66 was provided from local textile industry. Congo red dye was obtained from Alfa Aeser, India, MWt: 696.7 Da and Molecular fomula C_32_H_22_N_6_Na_2_O_6_S_2_ (chemical structure are shown below, Fig. 1). Nanofil Clay (NaMMT, Na_0.2_Ca_0.1_Al_2_Si_4_O_10_(OH)_2_(H_2_O)_10_) was supplied from Germany Commercial. Formic acid 85%, Hdrochloric acid (HCl) 37% and Sodium hydroxide (NaOH) ≥ 99% were supplied from local resource.

### Nylon sheet (NS) preparation

The adopted techniques for preparation of NS, was phase inversion method; a predetermined weight of nylon fibers waste was dissolved in 85% formic acid to prepare a 20% by weight nylon solution. The polymer solution is then casted into a dry and clean glass plate using film applicator to get NS of 0.2 mm thickness. The casted solution was dipped into a distilled water bath as a coagulation medium for 24 h, the separated NS was air dried for characterization and application.

### Nylon fiber sheet characterization

ATR-IR with range (400–4000 cm^−1^) was carried out using ALPHA II BRUKER, USA. Surface topography of the NS was investigated using FESEM QUANTA 250, Japan. The surface area of the prepared sheet was assessed by Brunauer–Emmett–Teller (BET) method by determination of nitrogen adsorption isotherm at 77 K via the Belsorp adsorption automatic specific surface area analyzer (Microtrac-BET, Japan). Wide-angle X-ray diffraction (WAXD) was used to analysis the crystallinity of the obtained sheet over a 2θ range of 5°–50° using Bruker D8 Advance ECO diffractometer using reflection mode.

### Contact angle

The contact angle was assessed using a digital camera connected to computer. A drop of deionized water (5 µL) was carefully delivered onto the sheet surface at room temperature (≃25 °C) and the angle among sheet-water interface and water–air interface was measured as an indication of sheet hydrophilicity.

### Assessment of point of zero charge (pHpzc) of the NS

The nylon fiber sheet’s point of zero charge (pH pzc) was identified utilizing the pH drift approach^30,31^. Briefly, 0.1 M HCl or 0.1 M NaOH was used to get the pH of 7% KCl to a range between 4 and 10. The pH-adjusted solution was combined with 0.025 g of NS, and the mixture was allowed to equilibrate for 24 h. The final pH was measured, and the initial pH is being plotted against the difference between the final pH and initial pH (pH final–pH initial). The pH pzc is the value at which the curve crosses the zero x-axis.

### Swelling percentage

The nylon sheets welling percentage was estimated by immersing determined weight of NS in pure water at ± 25 °C for about 24 h to attain swelling equilibrium^32^. After that, the NS was pulled out from the water, specked with filter paper and weighed. The % of swelling was measured by Eq. (1):

1 $$\mathrm{Swelling \% }=\frac{W2-W1}{W1}\times 100$$

where W_1_ (g) and W_2_ (g) are the weights of dried and swelled NS, respectively.

### Porosity properties

The porosity of the NS was estimated by the dry wet weight method^33,34^. The wet weight of the NS with definite dimension was measured after discarding of the excess water. Afterwards, the wet NS was air dried for 24 h and the dry weight was determined. Finally, the NS porosity was evaluated using Eq. (2):

2 $$Porosity \%=\left[\frac{\left[W2-W1\right]}{DWAh}\right] \times 100$$

where W_1_ (g) and W_2_ (g) are the weights of dried and swollen NS, respectively.

DW is the water density (0.998 g/cm^3^), A is NS area in the swelled state (cm^2^), and h is the thickness of NS in the wet state (cm).

### Adsorption and optimization experiments

Nylon sheet of 20% by mass based on fiber waste weight was employed as adsorbent material of anionic dye, Congo red (CR). Congo red dye solution have been prepared with concentration ranging from 100 to 700 mg/L. Then 10 ml of the different dye solutions is added to a predetermined weight of NS in a 50 ml glass flask. Then 50 rpm automatic shaker was used for variant times. The final concentration after adsorption was assessed using UV-Lambda 35 Perkin Elmer at λ_max_ = 495 nm.

The adsorption capacity q (mg g^−1^) for Congo red dye can be evaluated using the following Eq. (3)^35^.

3 $${q}_{e}=\left( {C}_{0}-{C}_{e}\right)\times V/m$$

where C_0_ and C_e_ in (mg/L), are the initial and equilibrium dye concentration, respectively, V in (L) is the volume of CR dye and m in (g) is the weight of NS adsorbent.

The removal efficiency for certain dye concentration at equilibrium can be explored by the next Eq. (4)^36^.

4 $$\%R=\frac{\left( {C}_{0}-{C}_{e}\right)}{{C}_{0 }}\times 100$$

Effects of pH and regeneration studies on adsorption capacity of NS for Congo red were studied.

### Adsorption isotherm studies

Different adsorption isotherms such as Langmuir, Freundlich, Temkin and Dubinin-radushkevic models isotherms were applied. The mathematical linear adsorption equations are shown by Eqs. (5, 7, 8, 11)^37,38^.

5 $$\frac{{C}_{e}}{{q}_{e}}=\frac{1}{{q}_{max}{k}_{l}}+\frac{{C}_{e}}{{q}_{max}}$$

where ${k}_{l}$, is the Langmuir constant that correlated to adsorption–desorption capability and q_max_ is the maximum adsorption capacity at full saturation of NS adsorbent, Ce in (mg/L) and q_e_ in (mg/g) are the dye concentration and adsorption capacity both at equilibrium, respectively.

Separation factor or equilibrium factor (*R*_L_) derived from Langmuir isotherm is unitless equilibrium factor can be displayed using the following equation (Eq. 6).

6 $${\text{R}}_{{\text{L}}} = { 1}/\left( {{1} + {\text{K}}_{{\text{l}}} {\text{C}}_{0 } } \right)$$

where, C_o_ is the starting dye’s concentration.

The Freundlich isotherm is shown in the following equation

7 $${\text{log}}{q}_{e}={\text{log}}K+\frac{1}{n}{\text{log}}{C}_{e}$$

where k is the Freundlich constant related to adsorption capacity and *n* is the empirical constant related to the adsorption affinity.

The linear equation of D–R isotherm can be represented in the following Eq. (8)

8 $${\text{ln}}qe={\text{ln}}{q}_{m}-(\beta {\varepsilon }^{2})$$

where q_m_ is the theoretical isotherm saturation capacity (mg/g), β (mol^2^/KJ^2^) represents the adsorption energy constant and $\varepsilon$ (kJ/mol) is the Polanyi potential identified with equilibrium and can be determined using the next Eq. (9).

9 $$\varepsilon =RT ln \bigg(1+\frac{1}{Ce}\bigg)$$

where R represents the universal gas constant (8.314 J/mol K) and T (K) is the temperature.

Then, the mean adsorption energy of E (KJ/mol) can be calculated for each adsorbent molecule as shown in Eq. (10):

10 $$E=1/\surd 2\beta$$

Temkin isotherm can be represented using the Eqs. (11, 12).

11 $$qe=B ln {k}_{t}+B{\text{ln}}{C}_{e}$$

12 $$B=\bigg(\frac{RT}{{b}_{t}}\bigg)$$

whereas b_t_ is the Temkin constant and K_t_ (L/mg)is constant at equilibrium binding.

### Kinetic studies

To study the rate of dye adsorption, pseudo first order and pseudo second order are used, in addition to Intra-particle diffusion and Elovich models. The linear mathematical models are shown in Eqs. (13–16)^39,40^, respectively.

13 $${\text{log}}\left({q}_{e}-{q}_{t}\right)={\text{log}}{q}_{e}-{k}_{1}*\frac{t}{2.303}$$

where, q_t_ is the adsorption capacity at time t (mg/g), and k_1_ is the rate constant per minute. A straight-line plot can be used which has a slope of (− k_1_/2.303) and an intercept of log (q_e_)

14 $$\frac{t}{{q}_{t}}=\frac{1}{{k}_{2}{q}_{e}^{2}}+\frac{1}{{q}_{e}}*t$$

where K_2_ is the equilibrium rate constant (g/mg min), a straight-line plot between t/qt and t give a slope of (1/q_e_) and an intercept of (1/K_2_q_e_^2^).

Intra-particle diffusion model can be represented using the following equation

15 $${q}_{t}={K}_{i} {t}^{1/2}+C$$

where K_i_ is intraparticle diffusion rate constant (mg/g min^1/2^) and C is constant represent the thickness of boundary layer, K_i_ and C were calculated from slope and intercept respectively, by plotting q_t_ versus t^0.5^.

Elovich model can be represented as the follow

16 $${q}_{t}=\frac{1}{\beta }{\text{ln}}\alpha \beta +\frac{1}{\beta }lnt$$

where $\alpha$(mg/g min) is the initial adsorption rate and $\beta$ (g/mg) is desorption constant. These correlated parameters were calculated from the linear plot of q_t_ against ln(t).

### Response surface methodology (RSM)

RSM is a powerful statistical tool used to optimize processes, it has the capability of developing representative models that relates outputs to different independent variables which is essential to reduce operational costs and minimize human resource consumption^41–43^. Box Behnken is one the most used response surface design in the field of chemistry^44,45^. In this study, the effect of incorporation of a predetermined percentage of NanoFil clay as a filler into NS will be studied with respect to percentage removal of Congo red dye.

## Result and discussion

### ATR-FTIR of nylon sheet (NS)

**Figure 2:**
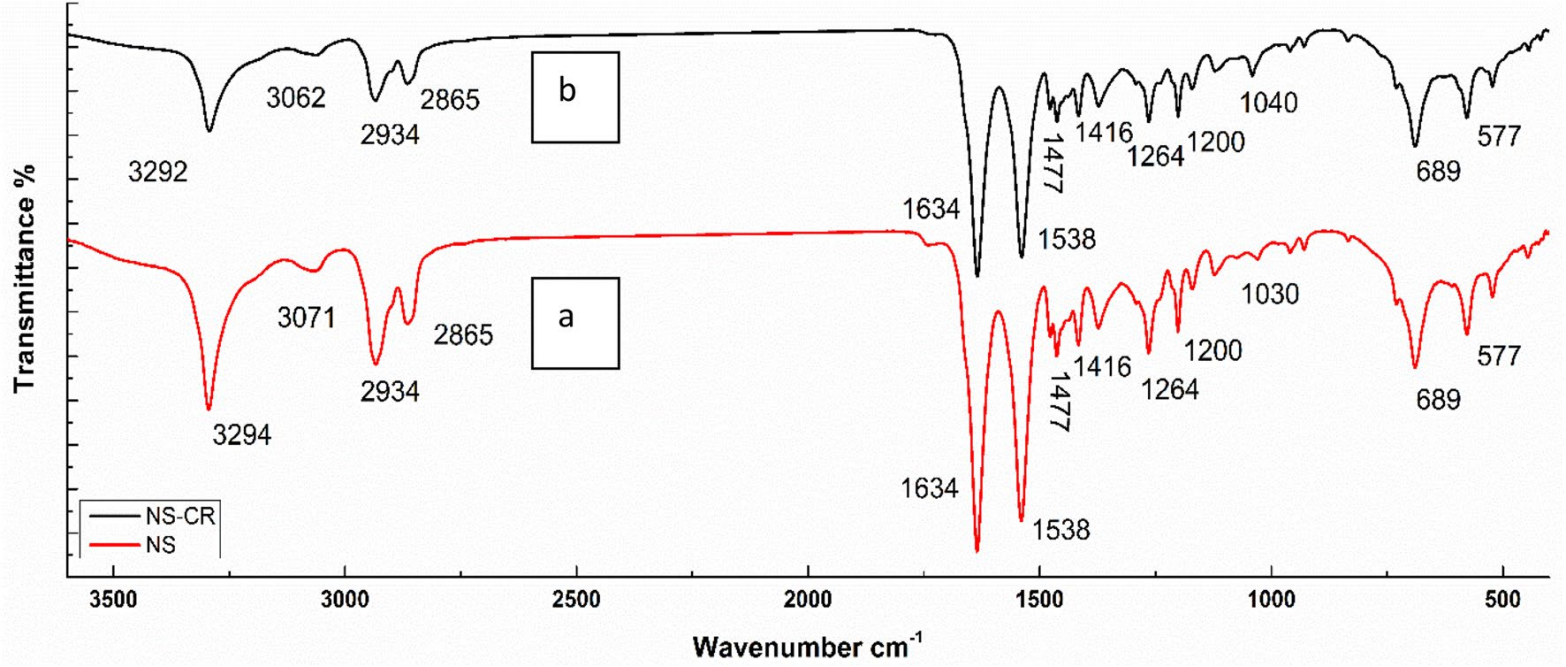
ATR-FTIR of NS (**a**) and NS-Congo red (**b**).

Nylon sheet is mainly composed of the following backbone structure [NH CO (CH_2_)x]. Figure 2 shows ATR-FTIR of NS, and NS-CR the following characteristic peaks were assigned to NS. At 3294 assigned to NH stretching of amide group, at 2933 and 2865 cm^−1^ assigned to CH_2_ asymmetric and symmetric vibration, respectively. Strong peaks appear at 1634 and 1538 assigned to amide carbonyl (C=O) stretching and C–N stretching, respectively^46–48^. Peaks at 1477, 1264, 1200, 689 and 577 attributed to –NH deformation, amide III stretching, CH_2_ twisting, C–C bending and C–C deformation, respectively^49^. The NS after Congo red adsorption showed the same characteristics peaks with less peak intensities and slight shift in few peaks this may be attributed to Congo red interaction with nylon sheet through electrostatic interaction and hydrogen bond formation.

### NS surface characterization

**Figure 3:**
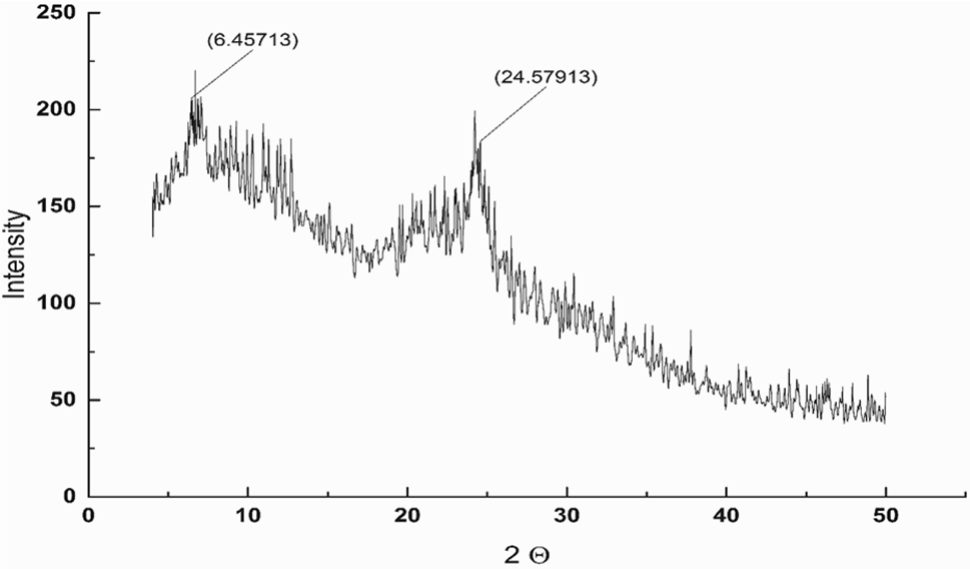
XRD pattern of NS.

Nylon fiber may be exist in two crystalline forms α or γ. XRD pattern of NS as shown in Fig. 3, show a sharp peak at 2θ = 24.5° (001 + 200 + 201), which correspond to γ form^50^.

**Figure 4:**
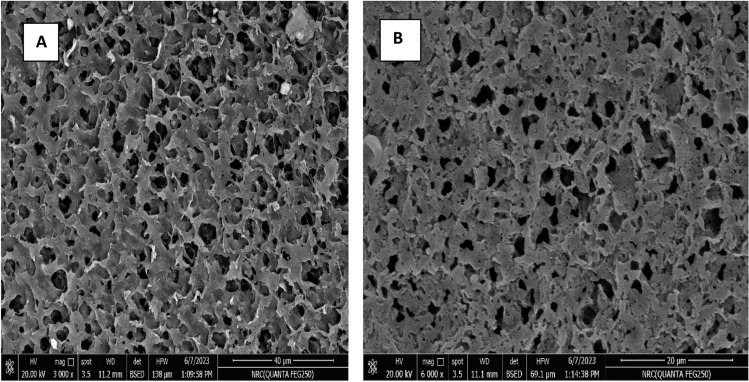
(**A**,**B**) SEM images with different magnifications of NS.

Figure 4A,B showed SEM images with different magnifications of NS and reveals the microporous structure of NS, with a homogenous void volume distribution which allows a homogenous penetration of CR molecules, thus allow for hydrogen bond interaction with actives sites of NS^51^.

**Figure 5:**
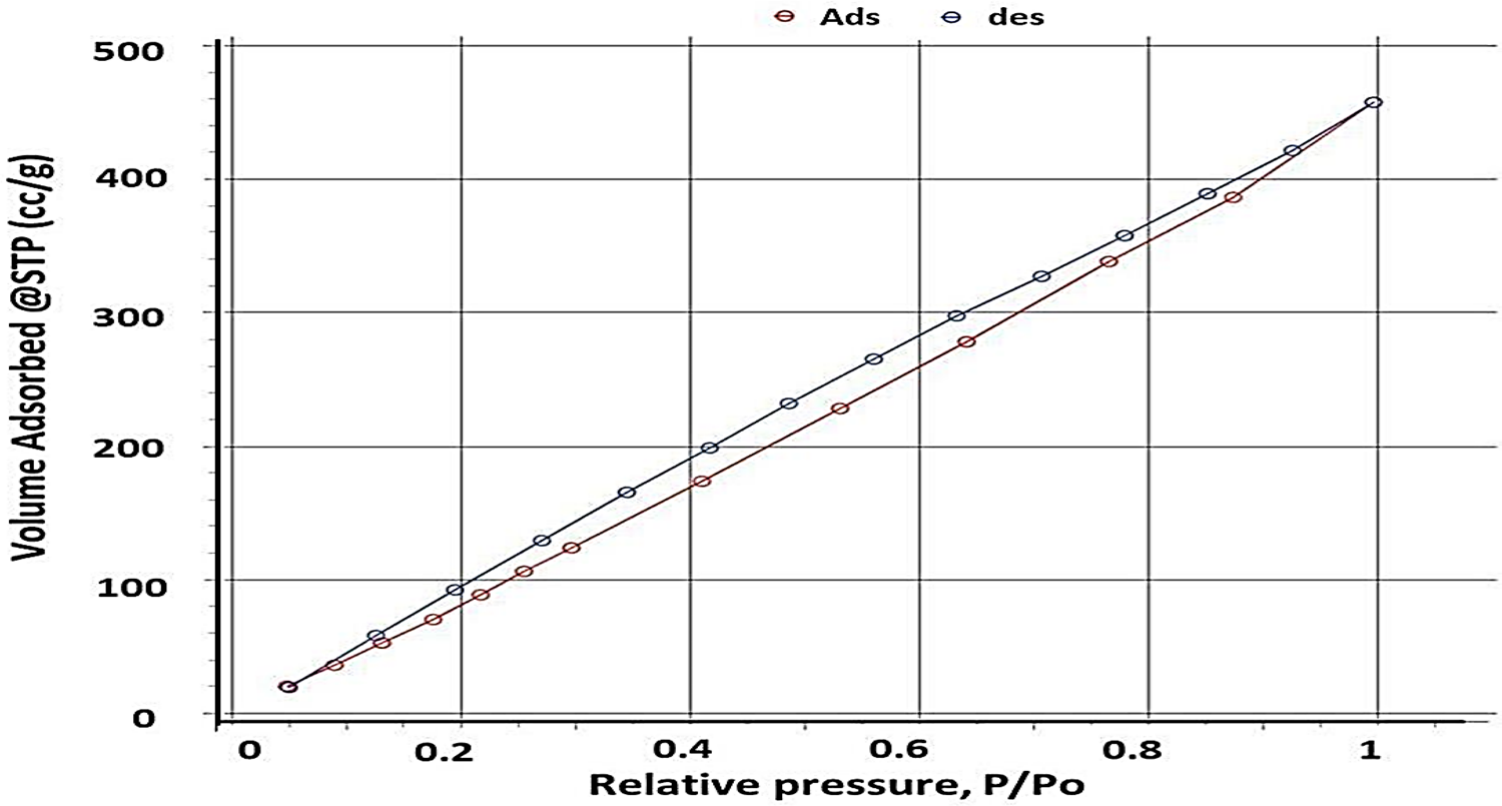
BET adsorption–desorption isotherm of NS.

As previously reported the surface area and pore volume have significant effect on enhancing adsorption efficiency of pollutants^52^. BET of NS is shown in Fig. 5 and tabulated in Table 1. From BET, NS has a high surface area of 767 (m^2^/g), which indicates a prominent material for adsorption. On the other hand, nitrogen adsorption–desorption isotherm exhibits type IV, also average pore volume and half pore width of NS reveal the meso-porous structure of NS^53,54^. The topography SEM and BET are consistent with the porosity % that estimated to be 66.5% using dry wet method.

**Table 1:** BET surface area and porosity/swelling % of the NS.

Adsorbent	BET surface area (m^2^/g)	Avg. pore radius (nm)	Half-pore width (nm)	Pore volume (cc/g)	Porosity %	Swelling %
NS	767	1.92	2.94	0.61	66.5	195

**Figure 6:**
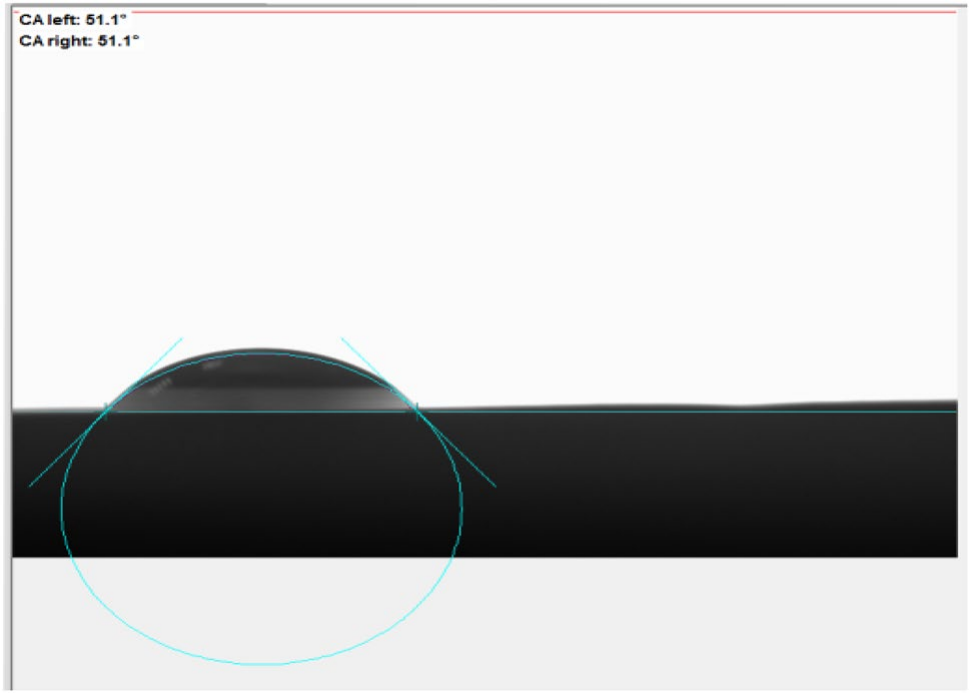
Contact angle of NS.

Hydrophilic nature of NS was confirmed via contact angle measurement, low water contact angles ranged [0°–90°] are preferred for high membrane hydrophilicity which was attributed to amide group founded in backbone structure of NS. The value of contact angle was found to be 51.1^o^ as shown below in Fig. 6. In addition, swelling % was measured to be 195% that indicates hydrophilicity nature of NS.

### Point of zero charge of NS (pHpzc)

**Figure 7:**
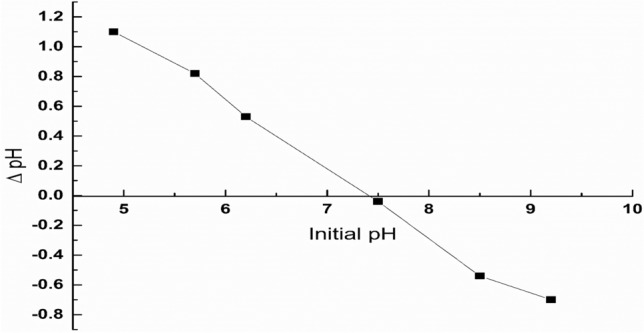
Point of zero charge of NS.

As illustrated in Fig. 7 the pH value of 7.3 was determined to be the point at which the net charge of the adsorbent NS is zero. Thus pH values lower than 7.3, where the surface becomes positively charged will enhance the electrostatic interaction with the anionic CR dye, would be favored for the adsorption of Congo red dye onto the adsorbent NS^30^.

### Adsorption and optimization experiments

**Figure 8:**
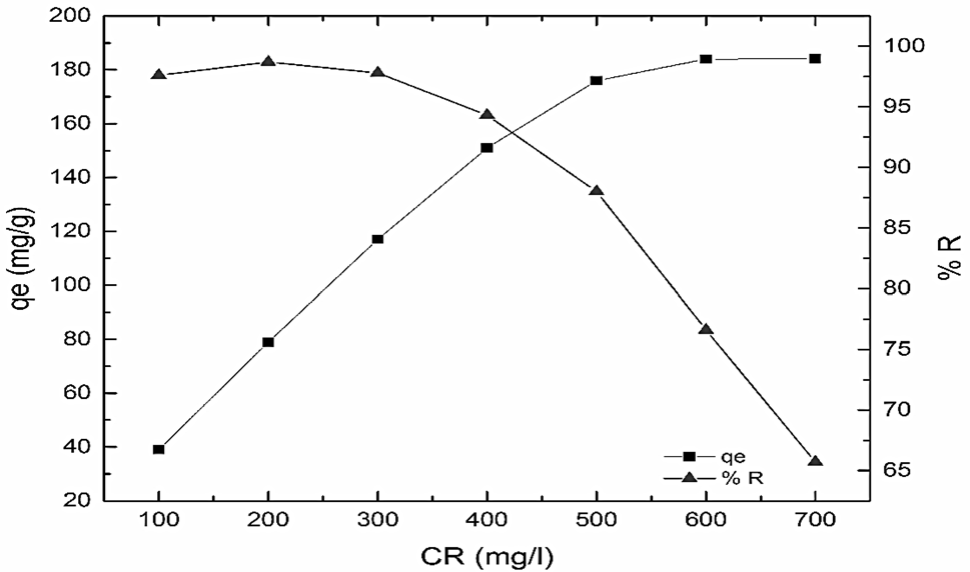
Effect of dye concentration on adsorption capacity and % R of NS (pH 6, 24 h).

To study the experimental equilibrium capacity of NS, (Fig. 8) illustrates the effect of a series of CR concentrations with equilibrium capacity and % R. The experimental equilibrium capacity qe_exp_ was attained at 600 mg/L of CR dye of value 188 mg/g, approximately. The NS adsorbent material exhibits a prominent % R ranged 99% to 95% for CR concentration ranged 100–400 mg/L.

**Figure 9:**
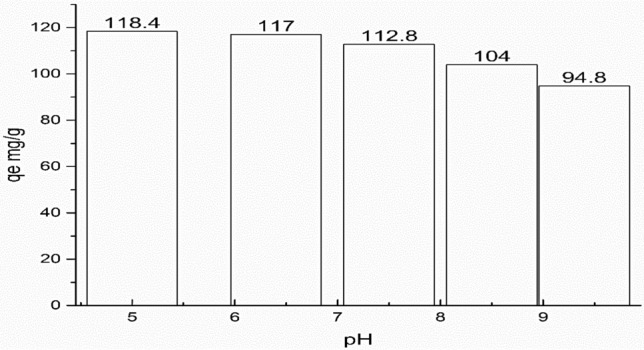
Effect of pH value of CR solution on the adsorption capacity of NS (CR Conc.: 300 mg/L, 24 h).

pH variation effect on adsorption capacity of CR of initial concentration 300 mg/L at 24-contact hour was illustrated in Fig. 9. As previously reported the adsorption of anionic CR dye most favorable in acidic medium, this because in acidic medium the abundant of positive charge enhance the electrostatic attraction between CR and NS. At pH 5 the q_eq_ (mg/g) was found to be 118.4, while at a higher pH round 9.5 the q_eq_(mg/g) was suppressed to be 94.8. This was attributed to a high negatively charge that promotes electrostatic repulsion between NS ad CR. However, at more acidic condition, the Congo red dye was turned into violet then blue color and afterwards it precipitated at acidic condition around pH 3. This observation was due to; at relatively high concentration of CR at acidic condition the hydrophobic interaction between the aromatic rings of the dye molecules was promote, which occurred by the π-π stacking phenomenon resulted in agglomeration of this dye at these condition^55,56^.

### Adsorption isotherms

**Figure 10:**
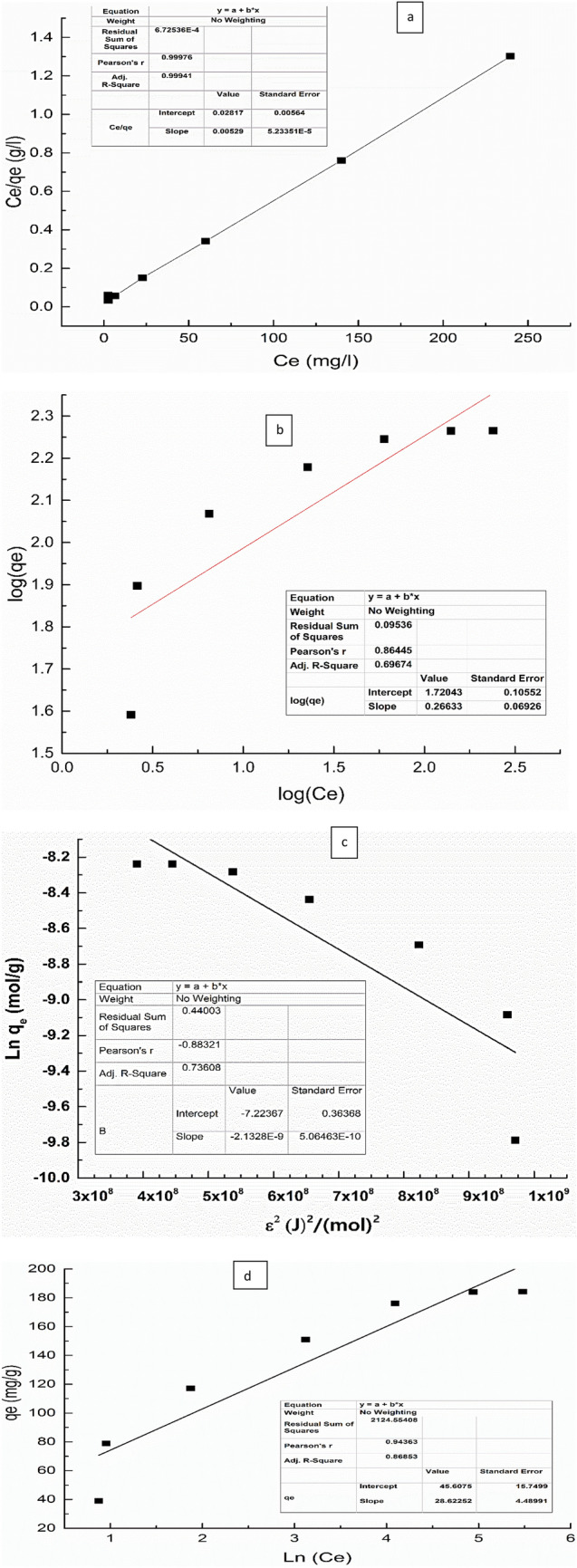
(**a**) Langmuir, (**b**) Freundlich, (**c**) D-R and (**D**) Temkin isotherms for CR dye adsorption by NS.

The adsorbent adsorbate interaction affinity was studied using different adsorption isotherms such as Langmuir, Freundlich, D-R and Temkin linear isotherms. A linear plot was displayed in Fig. 10 and isotherm parameters was shown in Table 2.

**Table 2:** Langmuir and Freundlich isotherms parameters for Congo red adsorption by NS.

	R^2^	K_L_(L/mg)	q _max_ (mg/g)	Linearized eqn	q_e exp_ (mg/L)
Lang	0.99	0.185	191.2	Y = 0.00529 x + 0.0282	188

	R^2^	n	K (mg/g)	Linearized eqn	
Frend	0.69	3.75	52	Y = 0.266 x + 1.72	

	R^2^	B (J/mol)	K_t_ (L/mg)	b_t_	Linearized eqn
Temkin	0.86	28.6	4.9	84.8	Y = 28.6 x + 45.6

	R^2^	q_m_ (mg/g)	E (KJ/mol)	Linearized eqn	
D-R	0.73	504	15.31	Y = − 2.13E^−9^ x − 7.2	

It was found that the adsorption characteristic of CR by NS obeyed Langmuir isotherm as it has higher R^2^ value than Freundlich isotherms and the value of calculated q_max_ is more close to the experimental one as displayed in Table 2. This means that NS may have homogenous adsorbent surface, and CR dye is completely adsorbed to the NS active sites by monolayer^57,58^. On the other hand, equilibrium factor (Rl) from Langmuir isotherm ranged from 0.051 to 0.0076 indicating favored Congo red adsorption onto the NS adsorbent substrate^59^.

For Dubinin–Radushkevic (D–R) isotherm; it is used to investigate the mechanism of adsorption either chemical or physical. The computed R^2^ value is 0.73 which proves that the D–R isotherm is not fitted for NS-CR adsorption. The calculated E from D–R is 15.3 kJ/mol. indicates chemical sorption process.

Temkin isotherm depend on assumption that the heat of adsorption would decrease by increasing the covering the adsorbent, the low R^2^ value of 0.86 indicated that Temkin isotherm is not valid model for CR-NS adsorption.

### Kinetic studies

**Figure 11:**
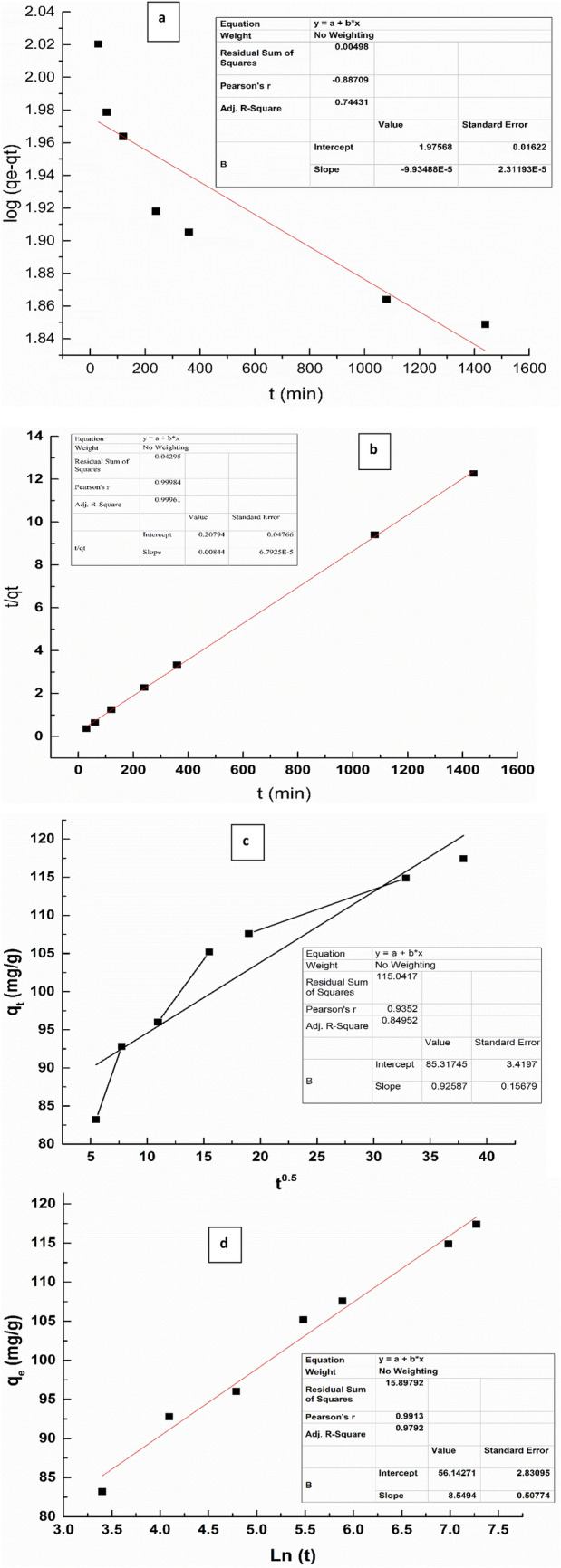
Pseudo first order (**a**), second order (**b**), intra-particle diffusion **(c)** and Elovich **(d)** kinetics models for CR adsorption by NS.

The adsorption of CR on NS was studied by different kinetics models as Pseudo first and second order Intra-particle diffusion and Elovich models. The linearized curves are shown in Fig. 11. The parameters are tabulated in Table 3. According to these data, pseudo second order model is more fitted due to high value of R^2^ of 0.999 as shown in Table 3. In addition to that, the calculated maximum adsorption capacity q_max_ was 118 for pseudo second order, which is close to experimental q_max_.

**Table 3:** kinetics models parameters for Congo red adsorption by NS.

	R^2^	qe (mg/g)	K1	Linearized eqn
Pseudo first order	0.74	93.3	0.000227	Y = − 0.000099 x + 1.97

	R^2^	qe (mg/g)	K2	Linearized eqn
Pseudo second order	0.99	118.4	0.000344	Y = 0.00844 x + 0.207

	R^2^	α (mg/g min)	β (g/mg)	Linearized eqn
Elovich	0.979	6.07E^3^	0.12	Y = 8.54 x + 56.14

	R^2^	Ki (mg/g min0.5)	C	Linearized eqn
Intra-particle diffusion	0.849	0.92	85.3	Y = 0.92 x + 85.3

Also, the R^2^ value of 0.97 of Elovich model support the chemisorption process. The low R^2^ of 0.849 for Intraparticle diffusion declares the unfitting for CR-NS adsorption system.

### Dye adsorption mechanisms

**Figure 12:**
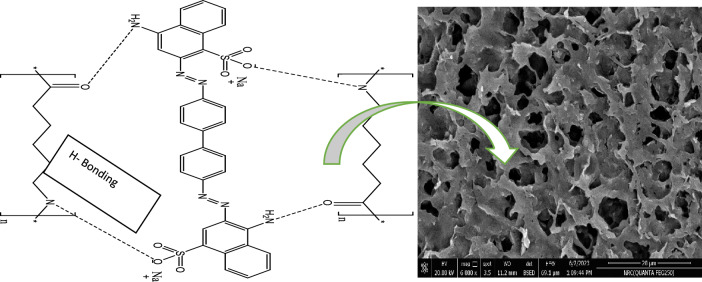
Proposed adsorption mechanism of CR-NS system.

As shown in Fig. 12, various mechanisms such as pore saturation, electrostatic interactions and hydrogen bonds can be involved in the remediation of CR using NS. As previously reported pores and porosity of the adsorbent was considered as one of the effective mechanisms in removing dyes. BET results showed that NS have a porous structure with micro-meso pore diameters. SEM topography also confirmed the existence of pores and in NS. These pores and grooves can be a place for adsorbing CR molecules (pore saturation mechanism)^60^. Another factor that can affect the adsorption process is the surface charges in the adsorbent and the electrostatic interactions as previously discussed in the pHzpc.). The functional groups in the NS and CR play an important role in their chemical affinity and in the removal efficiency.

NS and CR contain different functional groups such as –SO_3_, –C=O, –NH and –NH_2_ which can remove dye contaminants through hydrogen bonds^61^.

### Regeneration study

**Figure 13:**
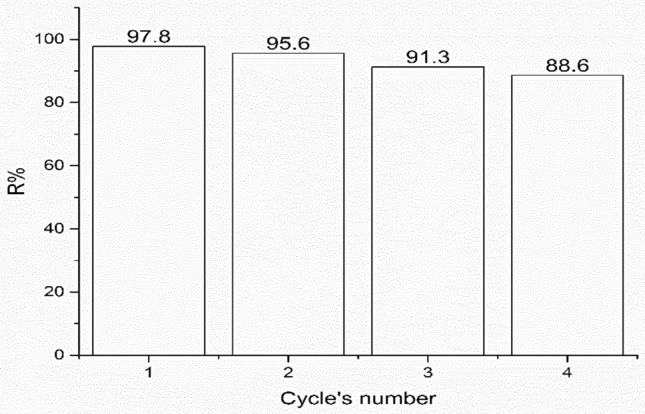
Adsorption–desorption studies of CR dye up to 4 successive cycles on NS with 10 ml dye solution; 0.025 g adsorbent dosage; initial pH of 6.5; 24 h of contact time; 300 mg/L of initial concentration of dye.

In order to evaluate the potential of NS for regeneration and reuse, desorption investigations were carried out in a batch design. The regeneration cycles was carried out via soaking the adsorbed NS in ethanol water mixture and a mechanical shaking process for 4 h to get the adsorbed dye out from NS matrices. As shown in Fig. 13, the results indicated that, the CR % removal after four rounds of subsequent recycling was determined to be 86.6%.The propensity of NS to exhibit noticeably high adsorption effectiveness after four adsorption–desorption cycles compared to other analogues polymeric adsorbent types, for examples acrylic fiber Ulva membrane exhibited 80% R after four regeneration cycles may be vital for the proper management of utilized adsorbent because it reduces the cost of the procedure^62^.

### Response surface methodology (RSM)

In the present study, the Box Behnken function of the Design-Expert® v.13.0.5 software program (Stat-Ease, Inc., USA) was utilized to investigate the interaction between three independent factors (Weight of fiber (%), Conc of clay (%) and Dose (g)) with respect to the dependent variable (Removal % of dye). The independent variables were ranged in predetermined amounts obtained from literature across three levels between 1 and + 1 dependent on the plan of the experimental matrix. The experiments and variables are shown in Table 4.

**Table 4:** Minimum and maximum values of the studied independent variables.

Variable	Minimum	Maximum
Fiber weight (%)	16	20
Clay concentration (%)	0	10
Adsorbent dose (g/l)	2.5	10

The removal efficiency was assessed and all the results were tabulated in Table 5.

**Table 5:** RSM variable results.

Run	Weight of fiber (%)	Conc of clay (%)	Dose (g/l)	Removal (%)
1	18	5	6.25	98.161
2	18	5	6.25	98.26
3	18	10	10	97.588
4	20	10	6.25	97.6
5	16	5	10	97.6
6	18	5	6.25	98.02
7	18	10	2.5	95.8
8	16	5	2.5	96.41
9	18	5	6.25	98.47
10	18	0	10	98.15
11	20	5	10	97.84
12	18	0	2.5	95.6
13	16	0	6.25	97.545
14	18	5	6.25	98.11
15	20	5	2.5	95.4
16	20	0	6.25	97.948
17	16	10	6.25	97.83

Regression equation was obtained using different models, the best statistical results were obtained when fitted to reduced cubic model, with R^2^ of 0.988 which indicates a strong model, the adjusted R^2^ and predicted R^2^ are 0.967 and 0.797 respectively, they are in agreement as they are within 0.2 of each other. The reduced cubic model results are shown in Table 6.

**Table 6:** Results of reduced cubic model.

Source	Sum of squares	df	Mean square	F-value	p-value	
Model	15.30	10	1.53	47.75	< 0.0001	Significant
A-weight of fiber	0.0075	1	0.0075	0.2335	0.6461	
B-conc of clay	0.0226	1	0.0226	0.7046	0.4334	
C-dose	7.94	1	7.94	247.66	< 0.0001	
AB	0.1002	1	0.1002	3.13	0.1275	
AC	0.3906	1	0.3906	12.19	0.0130	
BC	0.1452	1	0.1452	4.53	0.0774	
A^2^	0.2089	1	0.2089	6.52	0.0433	
B^2^	0.2647	1	0.2647	8.26	0.0283	
C^2^	5.75	1	5.75	179.55	< 0.0001	
AC^2^	0.1112	1	0.1112	3.47	0.1118	
Lack of fit	0.0738	2	0.0369	1.25	0.3794	Not significant

The Model F-value of 47.75 implies the model is significant. The P-values less than 0.0001 indicate model terms are significant and that the level of model is 99%. In this case C, AC, A^2^, B^2^, C^2^ are significant model terms. The Lack of Fit F-value of 1.25 implies the Lack of Fit is not significant relative to the pure error. Non-significant lack of fit is good as the model need to be fit.

The coefficient of determination of 98.8 supports that the rejection percent depends mainly on the independent variables. The standard deviation is 0.179, mean of the square is 97.43, and coefficient of variation is 0.1837 which is below 10% then the model is reproducible, and the value of AP statistic is 20.1 which is greater than 4, then the noise signal rate is sufficient^63^.

**Figure 14:**
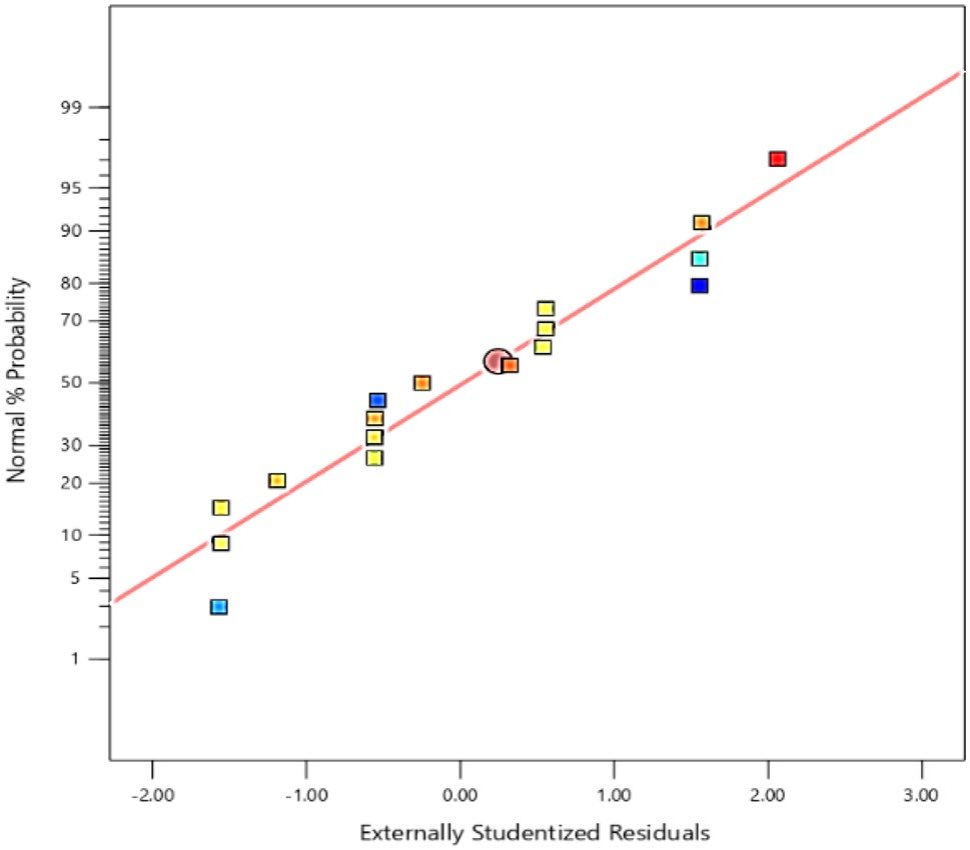
Normal plot of residuals.

**Figure 15:**
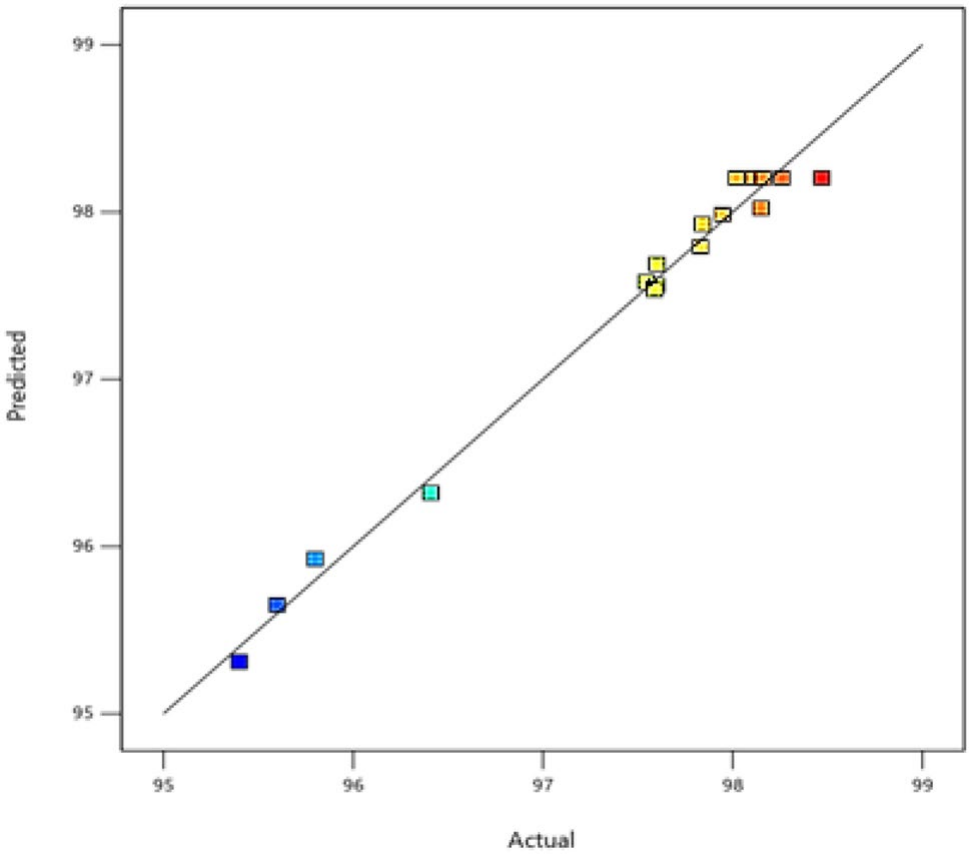
Predicted versus actual values.

The normal probability plot was used to check the normality of residuals, as shown in Fig. 14, all the points are nearly fitted to straight line which indicated a strong relationship between actual and predicted variables, on the other hand the adequate of this model is seen in Fig. 15 as the residuals are close to the diagonal line.

The model equation was found to be (Eq. 17):

17 $${\text{Y}}=-556.75{{\text{A}}}^{2}-100.28{{\text{B}}}^{2}-532{{\text{C}}}^{2}+174.38875{\text{A}}+10.028{\text{B}}+55.5612{\text{C}}+416.66{\text{AC}}+79.68802$$

where Y is removal percent, A is weight of fiber, B is Conc of clay, and C is dose of fiber.

#### Model optimization

**Figure 16:**
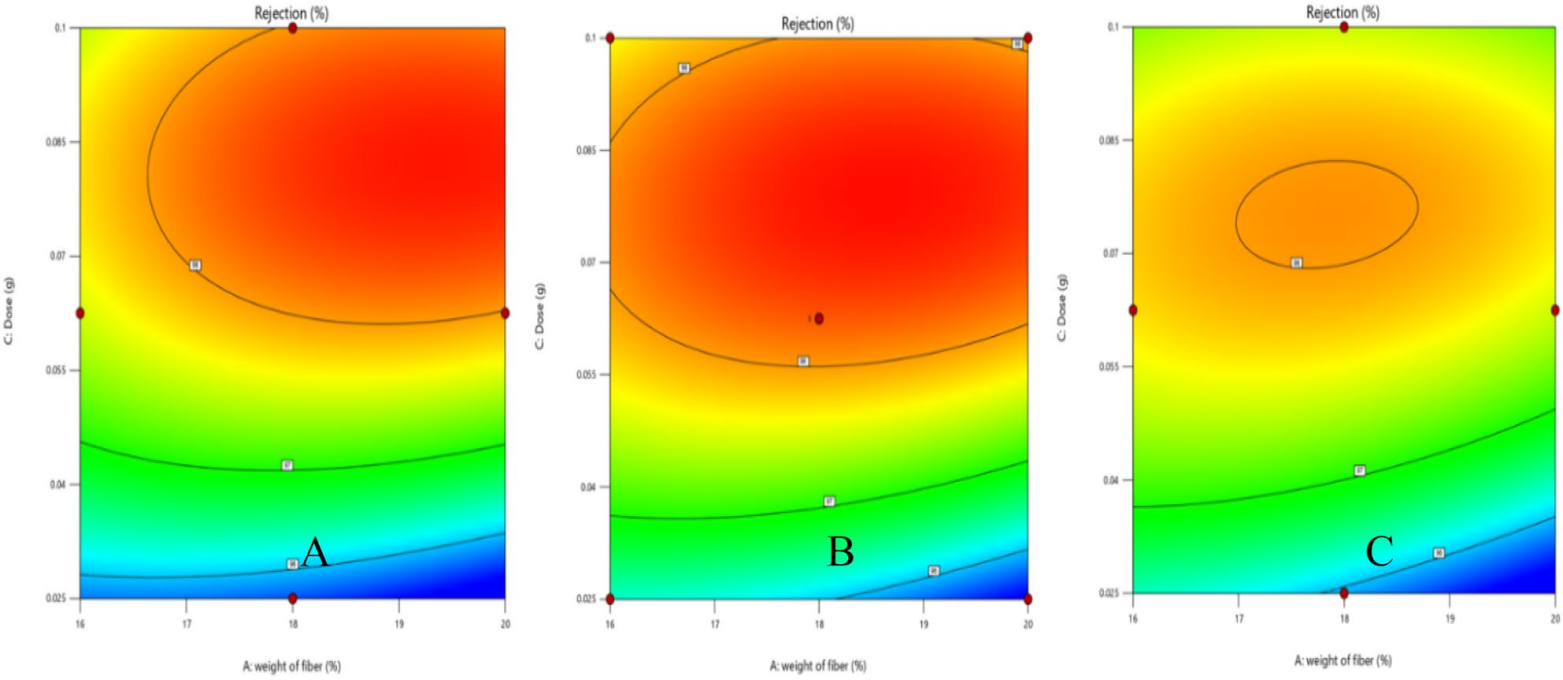
Interaction between fiber weight and dose at (**A**) minimum, (**B**) average, and (**C**) maximum values of clay.

**Figure 17:**
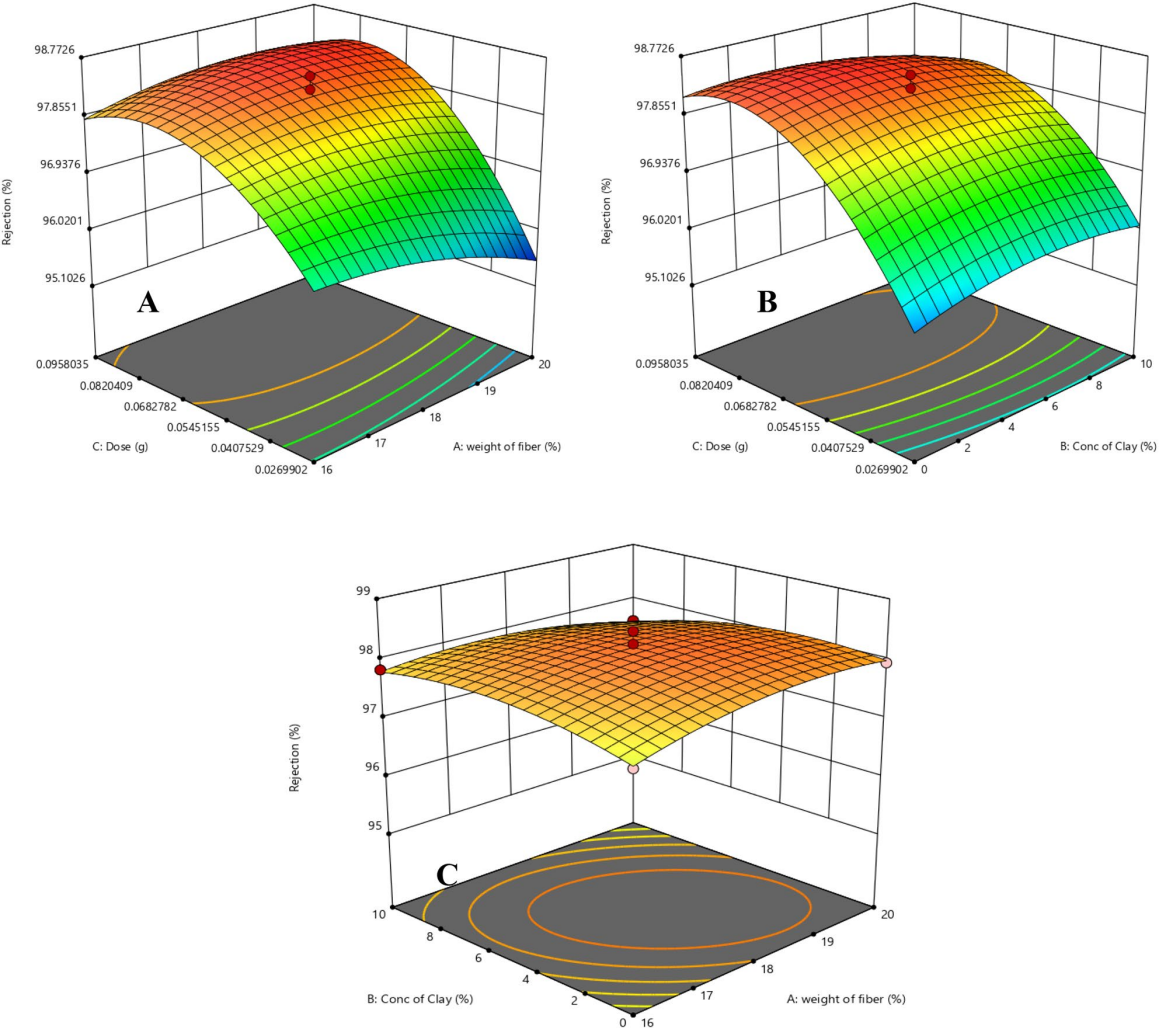
Interaction of (**A**) dose and weight of fiber, (**B**) dose and conc of clay, (**C**) conc of clay and weight of fiber.

Contours were used to assess the interactions between different parameters and their effect on the removal, the interaction between fiber weight and dose at minimum, average, and maximum values of clay is shown in Fig. 16. On the other hand, Fig. 17 illustrates the interaction between different variables, both of them reveals that minimum amount of clay is favored to obtain high removal percent, which means that the clay does not have significant effect for this model regarding the studied ranges of parameter.

**Figure 18:**
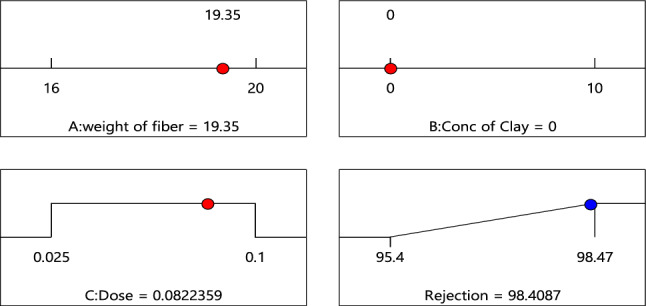
Optimum points predicted for the studied variables*.*

Optimization was used to find a value at which maximum desirable function is obtained. As shown in Fig. 18, 98.4% of removal could be achieved by using 19.35% wt. of fiber with 0.082g/10 ml dose and zero clay.

## Comparative study

A comparison between the present study with data in the literature related adsorption of CR on various adsorbent materials (Table 7). The table displayed the adsorption capacity of some waste materials as adsorbent for Congo red removal. These materials were modified chemically or physically through blending and mixing with functional active materials to be suitable for adsorption process. From the table, it can be detected that NS displayed prominent adsorption capacity compared to the reported adsorbents.

**Table 7:** Comparison of Congo red adsorption capacities for different adsorbents.

Adsorbent	q_max_ (mg/g)	References
Co_3_O_4_@SiO_2_ core/shell-nylon 6 magnetic nanocomposite	138.9	^64^
Acrylic fibers waste/nano‑chitosan	169.0	^25^
Carbon–metal LDOs based on plastic waste	317.0	^65^
AF-dried *Ulva fasciata*	30.9	^62^
Chinese yam peel-based adsorbent	86.6	^66^
Fly Ash	22.6	^67^
Aminated expanded polystyrene waste	1010.1	^68^
ZnO functionalized high silica zeolitic particles	161.3	^69^
Pineapple peel hydrogels	114.9	^70^
Nylon sheet (NS)	188.0	Current study

## Conclusion

The NS was prepared from nylon fiber waste via phase inversion technique and evaluated using SEM, XRD, FT-IR, BET, porosity and swelling test and contact angle. The NS is a prominent adsorbent for Congo red remediation from water with adsorption capacity of 188 mg/g at pH 6 and 24 h of contacting time. BET and SEM studies reveals the micro pours structure with a high surface area 767 m^2^/g. The adsorption capacity was enhanced in low pH media, whereas in basic and high pH media, the adsorption capacity decreased due to electrostatic repulsion. The main parameters affecting the adsorption capacity were initial CR concentration, pH and contact time. The equilibrium results showed that CR adsorption took place in a monolayer on NS and that Langmuir isotherms provided the best match for the data. Kinetic analyses showed that the data exhibited a pseudo-second-order behavior. Studies on regeneration revealed that NS was significantly stable for up to four cycles with 88% CR elimination. According to the findings, NS adsorbent is a promising and efficient adsorbent for the removal of CR from wastewater. The embedding of NanoFil clay into NS matrices was studied using RSM modeling technique. The finding reveals a non-significant effect on the adsorption capacity of NS for the studied parameters.

## Data Availability

Research data can be obtained from the corresponding author through email.
